# Adult stroke survivor’s reintegration to normal living: a scoping review protocol

**DOI:** 10.1186/s13643-021-01851-x

**Published:** 2021-11-22

**Authors:** Michael Opeoluwa Ogunlana, Pragashnie Govender, Olufemi Oyeleye Oyewole, Ifeoma Blessing Nwosu

**Affiliations:** 1 Federal Medical Centre Abeokuta, Abeokuta, Ogun State Nigeria; 2 College of Health Sciences, University of KwaZulu-Natal, Private Bag X54001, Durban, South Africa; 3 Discipline of Occupational Therapy, School of Health Sciences, University of KwaZulu-Natal, Private Bag X5400, Durban, South Africa; 4 Olabisi Onabanjo University Teaching Hospital, Sagamu, Ogun Nigeria; 5 Department of Medical Rehabilitation (Physiotherapy), Nnamdi Azikiwe University, Nnewi Campus, Awka, Anambra Nigeria

**Keywords:** Reintegration, Predictors, Normal living, Stroke survivors, Stroke, Community rehabilitation

## Abstract

**Background:**

To the best of our knowledge, a scoping review of the published literature investigating the determinants of adult stroke survivors’ reintegration to normal living has not been conducted. This scoping review aims to critically review the evidence investigating reintegration to normal living following a stroke. The following questions on reintegration to normal living after stroke will pivot this review: (i) what factors are associated with returning to normal living of stroke survivors? (ii) what are the overall determinants of reintegration to normal living of stroke survivors? To fully understand these questions, we also ask, how is reintegration to normal living assessed throughout stroke literature?

**Methods:**

A scoping review will be conducted based on the methodology presented by Arksey and O’Malley and extended by Levac and colleagues. The Preferred Reporting Items for Systematic Review and Meta-Analysis extension for scoping reviews (PRISMA-ScR) was adopted to develop the protocol. This study will include studies involving participants ≥ 18 years old, who are stroke survivors reintegrating to normal living in the community. With no time limitations, English language publications and all study designs reporting on reintegration to normal living of stroke survivors’ will be sourced. The abstract and full-text screening will be conducted by two independent reviewers, including data charting. Thematic analysis will be used to align relevant themes and will be presented in a narrative.

**Discussion:**

We anticipate that the scoping review will highlight the available resources and evidence on factors that determine reintegration to normal living of stroke survivors. This may contribute to informed empirical evidence for rehabilitation professionals to enhance the functional recovery of stroke survivors. It may also reveal other areas for research into reintegration to normal living for stroke survivors.

**Scoping review registration:**

The protocol has been registered prospectively on the Open Science Framework (https://osf.io/36tuz/).

Contributions to the literature

- Functional recovery for adult stroke survivors is dependent on several factors, and a few studies have documented the trajectories of return to normal living by stroke survivors.
- We propose a scoping review to critically review the evidence investigating reintegration to normal living following a stroke.
- The findings will serve as empirical documentation of the determinants of reintegration to normal living for adult stroke survivors.

## Background

Cerebrovascular accident or more commonly, stroke, is the second most common cause of death and disability worldwide. It is projected that the global stroke burden would increase from 38 million disability-adjusted life years in 1990 to 61 million in 2020 [1]. With increasing sophistication in the medical care of stroke incidents, there is a gradual increase in stroke survivors with stroke-related morbidities. These morbidities have necessitated increased utilization of physical rehabilitation facilities. Neurorehabilitation is often a long process that requires protracted periods of intervention both at inpatient and outpatient facilities. Care is often continued at home or community level to adapt the intervention to each survivor’s natural environment. Neuro-rehabilitation services are costly when available in the home environment. This has hindered the reintegration of stroke survivors into the community [2].

Reintegration to normal living is one of the most essential elements of stroke rehabilitation [3] and is defined as the ‘reorganization of physical, psychological and social characteristics so that the individual can resume well-readjusted living after incapacitating illness or trauma ’[4]. Reintegration to normal living is synonymous with functional status, which is the individual's typical performance [5]. Stroke survivors return to the mainstream of family, community life, engaging in normal roles, responsibilities, actively contributing to social groups, and the society is dependent on several factors [6–9]. Stroke survivors’ individual, physical, psychosocial, and environmental life domains are the main determinants of reintegration to normal living with depressive symptoms and perception of overall stroke recovery significantly affecting social reintegration [6, 10]. While the research by Obembe et al. [8] also revealed that post-stroke depression was significantly associated with reintegration to normal living, it was emphasized that motor recovery was equally important for community reintegration. Return to instrumental activity of daily living like driving a car is said to be significantly associated with reintegration to normal living by stroke survivors [11]. Engel-Yeger et al. [7] in a similar scoping review recommended the recovery of participation outcomes as necessary for stroke survivors’ functional recovery. Wesselhoff et al. [12] in a systematic review reported that stroke survivors’ community mobility was significantly decreased compared to persons without any neurological impairment. Community reintegration is a key reflection of participation and community mobility for stroke survivors; hence more recent research activities are concentrated on community reintegration. This review aims at critically reviewing the evidence investigating reintegration to normal living following a stroke. A scoping review methodology was selected towards identifying research areas around reintegration to normal living for stroke survivors using a range of research approaches and towards identifying the gaps in the current knowledge base to help guide future research in the field.

## Methods

This scoping review will be based on the framework outlined by Arksey and O’Malley [13], which include six iterative steps: (i) identifying the research question; (ii) searching for relevant studies; (iii) selecting the studies; (iv) charting the data; (v) collating, summarizing, and reporting the results; and (vi) consulting with stakeholders to inform or validate findings. The sixth step, namely consultation with relevant stakeholders, although a valued additional step, remains optional, and has been excluded from this review. Recommendations made by Levac and colleagues [14] will also be considered. The Preferred Reporting Items for Systematic Review and Meta-Analysis extension for protocols (PRISMA-P) [15] assisted in developing this protocol in the absence of a guideline for a scoping review protocol. Notwithstanding, the PRISMA extension for a scoping review (PRISMA-ScR) checklist will be followed to report this study [16]. This protocol has been registered on the Open Science Framework (https://osf.io/36tuz/).

### Step 1: Identifying the research question

This scoping review seeks to answer the following question: “To date, what evidence exists on adult stroke survivor’s reintegration to normal living?” The sub-questions for this review will include the following:

1. To date, what evidence is there on the determinants of reintegration of adult stroke survivors to normal living?
2. How are existing protocols, strategies, interventions available for adult stroke survivors assisting in their reintegration to normal living in their respective communities?
3. What are the gaps that exist in the reintegration of adult stroke survivors to normal living?

Table 1 illustrates the population, concept, and context of this proposed scoping review study.

**Table 1:** PCC framework for defining the eligibility of the studies for the primary research question

P: Population	Adult: These include individuals (stroke survivors) ≥ 18 years
C: Concept	Reintegration to normal living: Reintegration to normal living is one of the most important elements of stroke rehabilitation [3] and it involves ‘reorganization of physical, psychological and social characteristics so that the individual can resume well-readjusted living after incapacitating illness or trauma ’[4].
C: Context	Post-stroke rehabilitation: Community reintegration which is one of the main goals of rehabilitation [8] for stroke survivors after the acute phase of cerebrovascular accident is dependent on physical functioning and several interrelated facilitators and barriers in the social and physical environment [10].

### Step 2: Identifying relevant studies

#### Information sources

With support from a subject-librarian, the authors will conduct a systematic search within the following electronic databases: PubMed, Cumulated Index to Nursing and Allied Health Literature (CINAHL), Health Source: Nursing/Academic Edition, Web of Science, Scopus, and Google Scholar, for relevant published literature. This study will use a complete search strategy that employs keywords, medical subject headings (MeSH) or subject headings search terms that relate to key concepts, as well as Boolean terms “AND” and “OR”. A search strategy piloted in PubMed is presented in Table 2. A secondary search of relevant articles from the reference list of the included studies using a snowball approach will also be undertaken. To ensure reliability between reviewers, a series of training exercises will be conducted before the screening process. A single arbitrator/reviewer will resolve all discrepancies between reviewers by revisiting the inclusion and exclusion criteria and instituting an additional pilot test. EndNote reference manager will be used to compile all relevant articles as well as identify duplicate records.

**Table 2:** Pilot search in PubMed electronic database

Date	Keywords	Search results
23/06/2021	**Search: (((stroke) OR (cerebrovascular accident)) AND (community reintegration)) OR (community integration)** (("stroke"[MeSH Terms] OR "stroke"[All Fields] OR "strokes"[All Fields] OR "stroke s"[All Fields] OR ("stroke"[MeSH Terms] OR "stroke"[All Fields] OR ("cerebrovascular"[All Fields] AND "accident"[All Fields]) OR "cerebrovascular accident"[All Fields])) AND (("communal"[All Fields] OR "communalism"[All Fields] OR "communalities"[All Fields] OR "communality"[All Fields] OR "communally"[All Fields] OR "commune"[All Fields] OR "communes"[All Fields] OR "community s"[All Fields] OR "communitys"[All Fields] OR "residence characteristics"[MeSH Terms] OR ("residence"[All Fields] AND "characteristics"[All Fields]) OR "residence characteristics"[All Fields] OR "communities"[All Fields] OR "community"[All Fields]) AND ("reintegrate"[All Fields] OR "reintegrated"[All Fields] OR "reintegrating"[All Fields] OR "reintegration"[All Fields] OR "reintegrations"[All Fields] OR "reintegrative"[All Fields]))) OR ("community integration"[MeSH Terms] OR ("community"[All Fields] AND "integration"[All Fields]) OR "community integration"[All Fields])	14,534

### Step 3: Study selection

#### Eligibility criteria

This proposed study will include articles that meet the defined eligibility criteria as follows:

Inclusion criteria

This study will include the following:

- Studies involving participants ≥ 18 years old
- Articles reporting evidence on stroke survivors
- Articles reporting evidence on reintegration to normal living
- All study designs (interventional, observational, qualitative and mixed methods designs)
- English language publications and
- Articles published to the date of the last search in 2021

Exclusion criteria

This study will exclude the following:

- Opinion pieces and commentaries on reintegration to normal living of stroke survivors
- Review studies
- Articles reporting evidence involving individuals younger than 18 years old
- Articles published in languages other than English
- Full-text articles that cannot be found/accessed
- Study settings that included only hospital-level care

#### Selection process

**Fig. 1:**
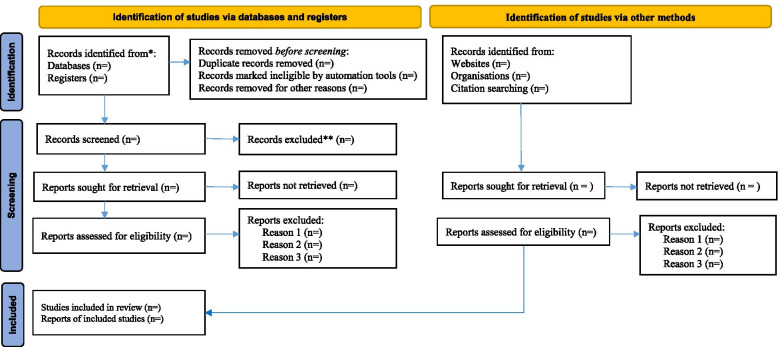
PRISMA flow diagram. *Consider, if feasible to do so, reporting the number of records identified from each database or register searched (rather than the total number across all databases/registers). **If automation tools were used, indicate how many records were excluded by a human and how many were excluded by automation tools. From: Page MJ, McKenzie JE, Bossuyt PM, Boutron I, Hoffmann TC, Mulrow CD, et al. The PRISMA 2020 statement: an updated guideline for reporting systematic reviews. BMJ 2021;372:n71. DOI: 10.1136/BMJ.n71. For more information, visit: http://www.prisma-statement.org/

A thorough title screening will be conducted by (MOO and IBN) in the electronic databases guided by the eligibility criteria. All relevant articles will be imported into an Endnote library and duplicates will be removed. Following this, the EndNote library will be shared among the review team for the next stage of the study selection process. A screening tool will be developed using the eligibility criteria for the abstract and full-text screening phases. Two reviewers (PG and MOO) will independently conduct abstract and full-text screening phases and group them into either an “include” or “exclude” category. Discrepancies between PG and MOO at the abstract screening phase will be addressed through a discussion by the review team until a consensus is reached. At the full-text phase, IBN and OOO will resolve any discrepancies between PG and MOO. Where an article could not be accessed freely online, assistance from the institution’s library services will be sought. The original authors will also be accessed via email for requests of full texts, if necessary. Cohen’s kappa coefficient (*κ*) statistic will be calculated to determine the inter-rater agreement between the reviewers at the end of the full-text screening phase. The PRISMA flow diagram [17] will be adopted to report the screening results as illustrated in Fig. 1 below.

### Step 4: Charting the data

A form will be developed in Google Forms for the data extraction and piloted to ensure its accuracy. PG and MOO will extract all relevant data from the included articles after a thorough reading of the full texts. The data extraction form will include the following details, namely, (i) title of the study, (ii) year of publication, (iii) study setting, (iv) aims and objective, (v) country of the study, (vi) study design, (vii) study participants, (viii) study results, (ix) findings relevant to answer the question, (x) conclusion, and (xi) recommendations. The form will be continually updated to enable the capturing of all relevant data to answer the review question.

### Step 5: Collating, summarizing, and reporting the results

The review team will ensure that the extracted data will be exposed to thematic analysis [18]. Relevant themes and sub-themes relating to the study objectives will be developed around the following: (i) determinants of reintegration, (ii) protocols, strategies and interventions available for stroke survivors that assist in community reintegration, and (iii) identified gaps in the successful reintegration of stroke survivors to their relevant communities. This will be presented as a narrative of the relevant themes and sub-themes. Where possible, tables and figures will also be used to present the results.

### Step 6: Methodological quality appraisal

The critical appraisal of evidence sources, although not mandatory, will be an included step in this review to assess the methodological quality using the mixed methods appraisal tool (MMAT) [19]. This instrument has a prescribed set of questions that examine the appropriateness of the different sections reported in each of the evidence sources. A quality score will be derived for each of the reported studies with a quality score of ≤ 50% interpreted as low quality, and a score ranging from 51 to 75% interpreted as average quality and a score ranging from 76 to 100% interpreted as high quality [20]. Two reviewers will independently conduct the quality appraisal to reduce bias.

#### Potential limitations to the scoping review

The inherent limitations of a scoping review are worth noting. First, we intend to focus and provide breadth rather than depth of information in this area of reintegration of stroke survivors to normal living via this review. Second, we will limit the included studies to published works only, and those disseminated in English, due to the vast number of potential studies as indicated in the pilot search.

## Discussion

The proposed scoping review aims to map the evidence on stroke survivors’ integration into community living. The extracted data will be presented in a tabular form in a manner to fulfil the aims of this scoping review. A descriptive summary will accompany the tabulated results to show how the results relate to the review's aims and objectives. The strengths and limitations of our scoping review method on the credibility of the results will be detailed. We anticipate that the scoping review will highlight the available resources and evidence on factors that determine reintegration to normal living of adult stroke survivors. This may contribute to an evidence-based interventional process for rehabilitation professionals to improve functional recovery in adult stroke survivors. It may also reveal further research areas into reintegration to normal living as it concerns adult stroke survivors.

## Data Availability

The datasets used and analysed during the current study will be available from the corresponding author on reasonable request.

## Abbreviations

MMAT: Mixed Methods Appraisal Tools
PRISMA: Preferred Reporting Items for Systematic Review and Meta-Analysis
PRISMA-P: Preferred Reporting Items for Systematic Review and Meta-Analysis extension for protocols
PRISMA-ScR: Preferred Reporting Items for Systematic Review and Meta-Analysis extension for scoping reviews
